# Serum levels of the novel adipokine isthmin-1 are associated with obesity in pubertal boys

**DOI:** 10.1007/s12519-022-00665-8

**Published:** 2023-01-03

**Authors:** Francisco Javier Ruiz-Ojeda, Augusto Anguita-Ruiz, Maria C. Rico, Rosaura Leis, Gloria Bueno, Luis A. Moreno, Mercedes Gil-Campos, Ángel Gil, Concepción M. Aguilera

**Affiliations:** 1 Department of Biochemistry and Molecular Biology II, School of Pharmacy, University of Granada, Granada, Spain; 2 Instituto de Investigación Biosanitaria Ibs.GRANADA, 18012 Granada, Spain; 3 Institute of Nutrition and Food Technology “José Mataix”, Center of Biomedical Research, University of Granada, Avda. del Conocimiento S/N. 18016 Armilla, Granada, Spain; 4 RG Adipocytes and Metabolism, Institute for Diabetes and Obesity, Helmholtz Center Munich, 85764 Munich, Germany; 5 Barcelona Institute for Global Health, ISGlobal, 08003 Barcelona, Spain; 6 CIBEROBN, (Physiopathology of Obesity and Nutrition CB12/03/30038), Institute of Health Carlos III (ISCIII), 28029 Madrid, Spain; 7 Unit of Investigation in Human Nutrition, Growth and Development of Galicia (GALINUT), University of Santiago de Compostela (USC), Santiago de Compostela, Spain; 8 Pediatric Nutrition Research Group, Institute of Sanitary Research of Santiago de Compostela (IDIS) CHUS-USC, 15706 Santiago de Compostela, Spain; 9 Unit of Pediatric Gastroenterology, Hepatology and Nutritio, Pediatric Service, University Clinical Hospital of Santiago (CHUS), 15706 Santiago de Compostela, Spain; 10 GENUD Research group, Institute of Sanitary Research of Aragón (IIS Aragón), University of Zaragoza, Zaragoza, Spain; 11 Instituto Agroalimentario de Aragón (IA2), Universidad de Zaragoza-CITA, Zaragoza, Spain; 12 Unit of Pediatric Endocrinology, University Clinical Hospital Lozano Blesa, 50009 Zaragoza, Spain; 13 Metabolism and Investigation Unit, Reina Sofia University Hospital, Maimónides Institute of Biomedicine Research of Córdoba (IMIBIC), University of Córdoba, 14071 Córdoba, Spain

**Keywords:** Children, Epigenetics, Isthmin-1, Obesity, Puberty

## Abstract

**Objectives:**

To evaluate whether there is an association between the serum levels of the novel insulin-like adipokine isthmin-1 (ISM1) and obesity-related phenotypes in a population of Spanish children and to investigate the plausible molecular alterations behind the alteration of the serum levels of this protein in children with obesity.

**Methods:**

The study population is a sub-cohort of the PUBMEP research project, consisting of a cross-sectional population of 119 pubertal children with overweight (17 boys, 19 girls), obesity (20 boys, 25 girls), and normal weight (17 boys, 21 girls). All subjects were classified into experimental groups according to their sex, obesity, and insulin resistance (IR) status. They were counted anthropometry, glucose and lipid metabolism, inflammation and cardiovascular biomarkers as well as isthmin-1 (ISM1) serum levels. This population was intended as a discovery population to elucidate the relationship between obesity and ISM1 levels in children. Furthermore, the study population had blood whole-genome DNA methylation examined, allowing deepening into the obesity–ISM1 molecular relationship.

**Results:**

Higher serum ISM1 levels were observed in boys with obesity than in normal weight (*P* = 0.004) and overweight (*P* = 0.007) boys. ISM1 serum levels were positively associated with body mass index (BMI) *Z-*score (*P* = 0.005) and fat mass (*P* = 0.058) and negatively associated with myeloperoxidase (MPO) (*P* = 0.043) in boys. Although we did not find associations between ISM1 serum levels and metabolic outcomes in girls, which may indicate a putative sexual dimorphism, fat mass was positively associated in all children, including boys and girls (*P* = 0.011). DNA methylation levels in two-enhancer-related CpG sites of ISM1 (cg03304641 and cg14269097) were associated with serum levels of ISM1 in children.

**Conclusions:**

ISM1 is associated with obesity in boys at the pubertal stage, elucidating how this protein might be of special relevance as a new biomarker of obesity in children. Further studies including a longitudinal design during puberty are needed.

**Supplementary Information:**

The online version contains supplementary material available at 10.1007/s12519-022-00665-8.

## Introduction

Childhood obesity is increasing globally, and total adiposity is the key driver of metabolic risk in children and adolescents, which represents a strong risk factor for insulin resistance (IR) and future type 2 diabetes (T2D) [1]. Puberty is a time of metabolic and hormonal changes, and it is associated with a reduced insulin sensitivity that recovers at puberty completion in only some children [2–4]. In growing children, adipocyte hypertrophy is associated with inflammation and local and systemic IR, independent of body mass index (BMI) and fat mass, with adipose tissue being essential to maintain functional metabolism [5]. However, the molecular mechanism of IR is still unknown, particularly in children. This is in part because processes such as growth and puberty affect insulin secretion and sensitivity [6]. Hence, understanding the molecular and biological processes underlying metabolic changes (glucose and lipid regulation) during puberty and identifying pathways and biomarkers that might help to increase peripheral glucose uptake would be beneficial to reduce the impact of obesity and to prevent T2D.

A recent paper reported that protein isthmin-1 (ISM1) is secreted by mature adipocytes and triggers a signaling cascade similar to that of insulin, stated as a novel adipokine that acts through an unidentified receptor tyrosine kinase and, at pharmacological doses in mice, ISM1 ameliorates metabolic disturbances associated with T2D, including hyperglycemia and liver steatosis [7, 8]. Currently, identifying non-invasive biomarkers such as this new adipokine offers a great chance for metabolic disease prevention. Serum levels of the novel insulin-like adipokine ISM1 are indeed associated with obesity in boys at the pubertal stage but not in girls in a well-characterized population of Spanish children under a cross-sectional design. Furthermore, we identified DNA methylation in two-enhancer-related CpG sites of the ISM1 region (cg14269097 and cg14269097) associated with serum ISM1 levels in children with obesity.

## Methods

This study was conducted within the context of the multicenter PUBMEP study “Puberty and metabolic risk in children with obesity”, which is a multicenter study recruited at different hospitals in Spain and previously published [9, 10]. Here, a sub-population of 119 pubertal children (54 boys and 65 girls) from the whole PUBMEP cohort were selected for analysis. The sub-population of children at the pubertal stage was selected carefully based on the other metabolic outcomes that were measured, including the epigenome-wide association studies (EWAs). A total of 38 children had normal weight (17 boys), 36 overweight (17 boys), and 45 children with obesity (20 boys). The children with following characteristics were excluded: birth weight < 2500 g; intake of any drug that could alter blood glucose, blood pressure or lipid metabolism; and not being able to comply with the study procedures and being participating or having participated in the last three months in an investigation project. This study was conducted according to the guidelines set out in the Declaration of Helsinki (Edinburgh 2000 revised), and all procedures were approved by the Ethics and Research Committee of Galicia Autonomous Community (2011/198 and 2016/522). Written consent was obtained from the parents of all the children. Anthropometric measurements, such as body weight (kg), fat mass (kg), fat-free mass (kg), height (cm), hip circumference (cm), and waist circumference (WC) (cm), were measured using standardized procedures. The BMI *Z*-score was calculated based on published Spanish reference standards. Blood pressure was measured three times for each individual by the same examiner using a mercury sphygmomanometer and following international recommendations [11]. Measures of lipid and glucose metabolism, hormones, and classical biochemical parameters were performed at the laboratories of each participating hospital following internationally accepted quality control protocols. Blood samples were collected under overnight fasting conditions and centrifuged, and plasma and serum were stored at −80 °C. The presence of IR in children was defined according to the homeostatic model assessment (HOMA) insulin resistance (HOMA-IR) index. The cutoff points were obtained from a previously well-described Spanish cohort composed of children and adolescents [12, 13]. The cutoff points for IR were based on the 95^th^ HOMA-IR percentile, considering sex (HOMA-IR ≥ 3.38 in boys and HOMA-IR ≥ 3.90 in girls). These cutoff points have already been tested and validated as good metabolic risk classifiers in our population according to the results from a previous PUBMEP report [10]. Plasma adipokines, inflammation, and cardiovascular risk biomarkers [adiponectin, leptin, resistin, tumor necrosis factor alpha (TNF-α), high-sensitivity C-reactive protein (hsCRP), interleukin (IL)-6, IL-8, total plasminogen activator inhibitor-1 (PAI-1), P-selectin, myeloperoxidase (MPO), monocyte chemoattractant protein-1 (MCP-1), soluble intercellular cell adhesion molecule-1 (sICAM-1), and soluble vascular cell adhesion molecule-1 (sVCAM)] were analyzed in all samples and time points using XMap technology (Luminex Corporation, Austin, TX) and human monoclonal antibodies (Milliplex Map Kit; Millipore, Billerica, MA) as previously reported [10, 14]. Descriptive data were expressed as the mean (standard deviation) or median (minimum–maximum) if not normally distributed. One-way ANOVA, Kruskal Wallis and the Welch test were employed to assess group differences. Genomic DNA was extracted from peripheral white blood cells, and DNA methylation analysis was carried out using the infinium methylation EPIC microarray using bead chip technology (Illumina, San Diego, CA, USA) as previously described [10]. ISM1 protein levels were determined in serum using human SEQ515Hu for ISM 1 (Cloud-Clone Corp., USA), an enzyme-linked immune absorbent assay kit, according to the manufacturers' instructions. The coefficient of variance was 4%. Two-way ANOVA and Tukey's multiple comparisons test were employed to assess group differences in ISM1 levels between boys and girls and normal weight, overweight, and obese children. Multiple linear regression (MLR) analyses were applied for all continuous variables to study their association with ISM1 levels. Confounders for analyses were selected based on expert knowledge and past experience [9, 10] for each group of outcomes analyzed. The general formula for the multiple linear models applied responded to Outcome ~ ISM1 + Sex + Age + Origin, which was modified depending on the outcome under study. For outcomes related to body composition (BMI *Z*-score and fat mass), models were further adjusted for insulin resistance index, so findings respond only to associations between ISM1 and body composition: Outcome ~ ISM1 + Sex + Age + Origin + HOMA. For outcomes related to glucose metabolism [e.g., glucose levels, HOMA, and quantitative insulin sensitivity check (QUICKI) indexes], models included BMI *Z*-score instead of HOMA to detect only association signals due to IR: Outcome ~ ISM1 + Sex + Age + Origin + BMI *Z*-score. For the rest of the outcomes under study (e.g., inflammatory and cardiovascular biomarkers), both BMI *Z*-score and HOMA were included as confounders: Outcome ~ ISM1 + Sex + Age + Origin + BMI *Z*-Score + HOMA. The variance inflation factor for all tested models and the correlation between cofounders were investigated to check for multicollinearity problems (Supplementary Table 1 and Supplementary Fig. 1). No correlation was found between adjusting covariates. A *P* value < 0.05 was considered significant. Given the number of analyzed outcomes, we considered the false discovery rate (FDR) as in Benjamini and Hochberg to correct for multiple hypothesis testing. MLRs were also applied for all calculated deltas to study their correlation with the change in ISM1 levels. All described analyses were performed in R environment version 3.6.0.

## Results

**Fig. 1:**
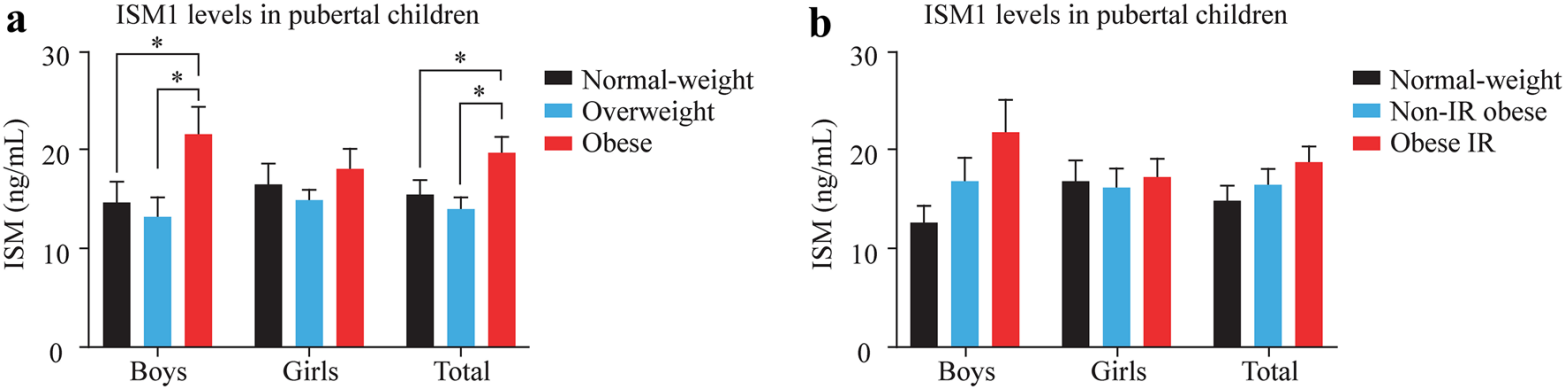
Group comparisons for ISM1 serum levels (ng/mL) in the pubertal population of 119 children. **a** Comparison between normal weight, overweight, and obese. **b** Comparison between normal weight non-IR, non-IR, and IR in children. Two-way ANOVA and Tukey's multiple comparisons test were employed to assess group differences in ISM1 levels according to standard statistical assumptions. *P < 0.05. *ISM1* isthmin-1, *IR* insulin resistance

The general characteristics of the 119 children in this cross-sectional study are shown in Supplementary Table 2. ISM1 serum levels according to sex are shown in Fig. 1a. Higher ISM1 serum levels were observed in boys with obesity when compared with normal weight (*P* = 0.004) and overweight (*P* = 0.007), un-adjusted. However, no changes were observed in girls. When all subjects of the sample were compared together, we found higher levels in children with obesity than in those with normal weight (*P* = 0.041) and those who were overweight (*P* = 0.010). A trend to increase ISM1 levels in boys with IR was observed, but no significant differences were shown between normal weight non-IR, non-IR, and IR children (Fig. 1b).

**Fig. 2:**
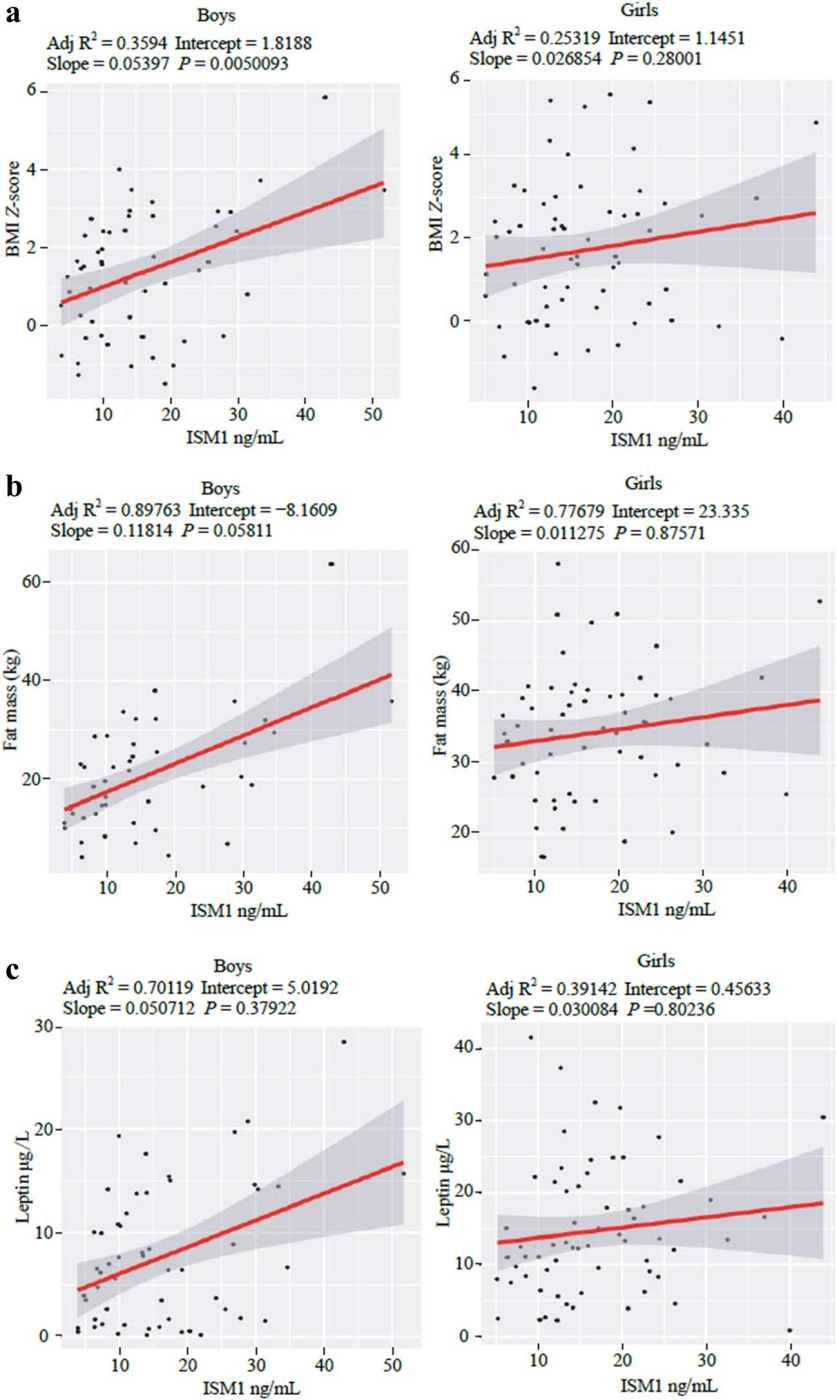
Multiple linear regression analyses between the change in ISM1 serum levels (ng/mL) and the changes in BMI Z-score, fat mass (kg), and leptin (µg/L) in the cross-sectional cohort. **a** The linear model with delta BMI Z-score as the dependent variable; **b** The model for delta fat mass as the dependent variable, and **c** The model for delta leptin as the dependent variable. *BMI* body mass index, *ISM1* isthmin-1

To elucidate the relationship between ISM1 and obesity, MLRs were further conducted in a wide range of metabolic outcomes separated by sex (Table 1) and properly adjusted by confounders such as age, origin, height, BMI, and IR when applicable. In boys, the strongest association was found for BMI *Z*-score (*P* = 0.005), but not in girls (Fig. 2a and Supplementary Fig. 1a). In addition, fat mass did not exhibit a significant association in girls (Fig. 2b); however, we observed a positive association with ISM1 serum levels in boys (*P* = 0.058) and all individuals (*P* = 0.011) (Fig. 2b and Supplementary Fig. 1b). Surprisingly, although both ISM1 and leptin levels were significantly higher in children with obesity, no significant associations were identified with leptin in either boys or girls (Fig. 2c and Supplementary Fig. 1c). On the other hand, MPO (*P* = 0.043) was inversely associated with ISM1 serum levels in boys. All of them were properly adjusted for confounders (Table 1) in both boys and girls.

**Table 1:** Association between the change in ISM1 serum levels and the change in metabolic outcomes in boys and girls in the cross-sectional population (PUBMEP study)

Outcome	Beta	SE	CI (low)	CI (high)	*T *value	*P *value	FDR
Boys							
BMI *Z*-score	0.3567	0.0183	0.0179	0.0899	2.9390	0.0050*	0.1352
MPO (µg/L)	−0.3498	2.8459	−1.4954	−0.3392	−2.0792	0.0435*	0.4482
Fat mass (kg)	0.1144	0.0605	−0.0005	0.2368	1.9508	0.0581	0.4482
SBP	0.2624	0.2514	−0.0471	0.9385	1772.56	0.0829	0.4482
AST (U/L)	0.2731	0.0660	−0.0177	0.2412	1.6909	0.0974	0.4482
ALT (U/L)	0.2352	0.1133	−0.0338	0.4106	1.6611	0.1031	0.4482
HsCRP (mg/L)	0.2343	0.3489	−0.1283	1.2393	1.5921	0.1182	0.4482
TAG (mg/dL)	0.2323	0.4040	−0.1739	1.4100	1.5296	0.1328	0.4482
Resistin (µg/L)	0.2239	0.1040	−0.0575	0.3503	1.4065	0.1659	0.4979
sICAM-1 (mg/L)	−0.2084	0.0098	−0.0315	0.0068	−1.2590	0.2141	0.5780
GGT (U/L)	0.1905	0.1046	−0.0811	0.3290	1.1845	0.2421	0.5943
DBP	0.1500	0.1634	−0.1569	0.4839	0.9999	0.3225	0.6495
MCP-1 (ng/L)	0.1624	0.6942	−0.6765	2.0450	0.9855	0.3292	0.6495
TNFα (ng/L)	0.1488	0.0122	−0.0128	0.0350	0.9075	0.3686	0.6495
Leptin (µg/L)	0.0797	0.0571	−0.0612	0.1627	0.8875	0.3792	0.6495
IL-6 (ng/L)	−0.1892	0.7887	−2.2416	0.8504	−0.8818	0.3848	0.6495
QUICKI	0.1228	0.0004	−0.0005	0.0012	0.8131	0.4200	0.6671
tPAI1 (µg/L)	−0.0826	0.1658	−0.4275	0.2225	−0.6180	0.5394	0.7742
P Selectine (µg/L)	−0.0903	0.2929	−0.7529	0.3955	−0.6098	0.5448	0.7742
HDL-c (mg/dL)	−0.0549	0.1622	−0.3829	0.2531	−0.4001	0.6908	0.8216
Hip circumference (cm)	−0.0269	0.0989	−0.2299	0.1581	−0.3626	0.7184	0.8216
IL-8 (ng/L)	−0.0565	0.0872	−0.2005	0.1416	−0.3368	0.7376	0.8216
Fat-free mass (kg)	−0.0475	0.1870	−0.4283	0.3047	−0.3307	0.7425	0.8216
Insulin (mU/L)	−0.0444	0.1177	−0.2668	0.1946	−0.3064	0.7605	0.8216
HOMA-IR	−0.0454	0.0269	−0.0611	0.0446	−0.3061	0.7607	0.8216
Adiponectin (mg/L)	0.0219	0.1034	−0.1863	0.2193	0.1593	0.8740	0.9077
Glucose (mg/dL)	−0.0016	0.1121	−0.2210	0.2185	−0.0109	0.9913	0.9913
Girls							
HsCRP (mg/L)	−0.2125	0.2759	−0.9938	0.0877	−1.6420	0.1061	0.9591
tPAI1 (µg/L)	−0.2035	0.2221	−0.7962	0.0746	−1.6238	0.1098	0.9591
Fat mass (Kg)	0.0855	0.0813	−0.0429	0.2758	1.4319	0.1590	0.9591
Fat-free mass (Kg)	0.1370	0.0821	−0.0489	0.2729	1.3644	0.1791	0.9591
AST (U/L)	−0.1610	0.0898	−0.2849	0.0673	−1.2108	0.2308	0.9591
BMI *Z*-score	0.1310	0.0246	−0.0214	0.0751	1.0903	0.2800	0.9591
Adiponectin (mg/L)	−0.1419	0.1153	−0.3500	0.1020	−1.0752	0.2867	0.9591
ALT (U/L)	−0.1225	0.0849	−0.2540	0.0788	−1.0313	0.3066	0.9591
GGT (U/L)	−0.1220	0.0689	−0.1991	0.0709	−0.9303	0.3560	0.9591
QUICKI	−0.0911	0.0004	−0.0012	0.0005	−0.7607	0.4498	0.9591
HDL-c (mg/dL)	0.0944	0.2280	−0.2786	0.6154	0.7381	0.4634	0.9591
HOMA-IR	0.0872	0.0345	−0.0428	0.0927	0.7205	0.4740	0.9591
Insulin (mU/L)	0.0751	0.1448	−0.1913	0.3762	0.6386	0.5254	0.9591
Resistin (µg/L)	0.0889	0.2005	−0.2674	0.5187	0.6264	0.5334	0.9591
DBP	0.1065	0.1772	−0.2105	0.4843	0.7723	0.4433	0.9591
MPO (µg/L)	0.0728	2.2843	−3.3294	5.6253	0.5025	0.6175	0.9591
IL6 (ng/L)	0.0768	0.6797	−1.0226	1.6420	0.4556	0.6510	0.9591
TNFα (ng/L)	−0.0585	0.0149	−0.0358	0.0228	−0.4329	0.6666	0.9591
Glucose (mg/dL)	0.0483	0.1534	−0.2477	0.3537	0.3452	0.7311	0.9591
sICAM-1 (mg/L)	−0.0374	0.0051	−0.0115	0.0088	−0.2589	0.7965	0.9591
Leptin (µg/L)	−0.0275	0.1196	−0.2645	0.2044	−0.2514	0.8023	0.9591
SBP	0.0402	0.1749	−0.3043	0.4120	0.2948	0.7692	0.9591
TAG (mg/dL)	0.0189	0.5974	−1.0837	1.2581	0.1459	0.8844	0.9591
IL-8 (ng/L)	−0.0207	0.0732	−0.1542	0.1329	−0.1452	0.8850	0.9591
Hip circumference (cm)	−0.0044	0.0948	−0.1931	0.1786	−0.0760	0.9396	0.9591
MCP-1 (ng/L)	−0.0081	0.7248	−1.4617	1.3798	−0.0565	0.9551	0.9591
P selectine (µg/L)	−0.0066	0.2215	−0.4455	0.4227	−0.0514	0.9591	0.9591

Multiple regression analyses with the change in ISM1 serum levels as independent variable. Models were adjusted for the change in BMI *Z*-score, the origin, and the pubertal stage reached. When the dependent variable was the change in the BMI *Z*-score, we replaced the BMI confounder with the change in insulin levels. The change in height was further included in models when the dependent variable was change in blood pressure. *ISM1* isthmin-1, *SE* standard error, *CI* confidence interval, *FDR* false discovery rate, *AST* aspartate aminotransferase, *ALT* alanine aminotransferase, *BMI* body mass index, *WC* waist circumference, *SBP* systolic blood pressure, *DBP* diastolic blood pressure, *HOMA-IR* homeostasis model assessment for insulin resistance, *QUICKI* quantitative insulin sensitivity check index, *TAG*, triglycerides, *HDL-c* high-density lipoproteins cholesterol, *hsCRP* high-sensitivity C-reactive protein, *MCP-1* monocyte chemoattractant protein-1, *GGT* gamma-glutamyl transferase, *TNF-α* tumor necrosis factor alpha, *TSH* thyroid-stimulating hormone, *IL* interleukin, *PAI-1* plasminogen activator inhibitor-1, *MPO* myeloperoxidase, *sICAM-1* soluble intercellular cell adhesion molecule-1. *P < 0.05

**Fig. 3:**
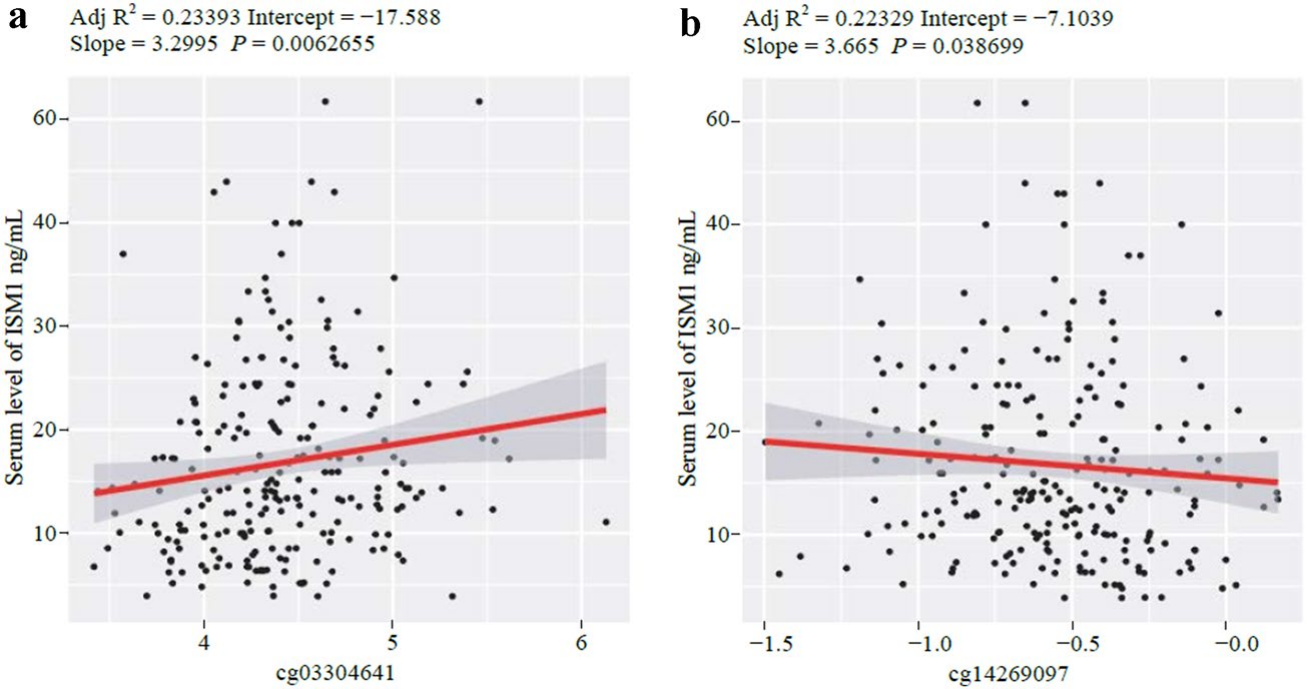
Multiple linear regression analyses between the change in *ISM1* DNA methylation status and ISM1 serum levels (ng/mL). **a** The linear model with delta ISM1 serum levels as the dependent variable and cg03304641 as the independent variable. **b** The linear model with delta ISM1 serum levels as the dependent variable and cg14269097 as the independent variable. *ISM1* isthmin-1, *Adj *adjusted

In recent years, CpG DNA methylation has been reported to be an epigenetic marker of cellular memory without changes in the DNA sequence, which is involved in numerous diseases and has been established as an important etiological molecular mechanism and a link with environmental exposures [15]. In obesity, previous studies have confirmed that the epigenome is an important regulator of gene expression [16]. Therefore, our research hypothesis was that epigenetic alterations in *ISM1* could be relevant for understanding its role in obesity. Totally 51 methylation sites were selected from the Infinium Methylation EPIC microarray, of which two were annotated as promoter-associated CpGs (Supplementary Table 3). All the CpGs were annotated as open sea. We found a positive significant association between the methylation status of the probe cg03304641 and ISM1 serum levels in pubertal children (*P* = 0.006) (Fig. 3a, Supplementary Fig. 2a and Supplementary Table 3). However, for the probe cg14269097, there was a negative association with ISM1 serum levels (*P* = 0.038) (Fig. 3b, Supplementary Fig. 2b and Supplementary Table 3).

## Discussion

In the present work, we show higher serum levels of the novel insulin-like adipokine ISM1 in pubertal children with obesity, with an association with BMI *Z*-score and fat mass in boys. Moreover, ISM1 was inversely associated with MPO, an enzyme associated with IR and inflammation in overweight individuals [17, 18]. These findings illustrate how this protein might be of special relevance as a new biomarker of obesity in children.

In obesity, hypertrophic adipocytes and adipose tissue-resident immune cells accelerate a low grade and chronic proinflammatory profile with altered secretion of adipokines, thereby exacerbating cardiometabolic disease [19]. In this context, the production and secretion of adipokines, which contribute to systemic energy metabolism by different mechanisms, are dependent on the energy status of adipose tissue. Hence, further preclinical and clinical studies exhibiting the activation or inhibition of the signaling of specific adipokines (e.g., using adipokine-neutralizing antibodies) may contribute to an approach suitable to treat or prevent the development of metabolic disease. Nevertheless, efficacy and safety in humans need to be confirmed [20].

ISM1 is a secreted protein that was originally discovered in fetal brain development and is expressed in the brain, lung, vasculature, skin, and immune cells [21]. ISM1 has been recently identified in mouse and human adipocytes, regulating glucose uptake while suppressing hepatic lipid synthesis, thus improving hyperglycemia and reducing lipid accumulation in mouse models. In addition, circulating plasma levels of human ISM1 have been detected at an average of 50 pg/mL and tend to positively correlate with BMI but not with glucose in female individuals [7]. We found a relationship between circulating ISM1 levels and obesity in pubertal children.

The motivation for focusing on the identification of new biomarkers in puberty lies in the fact that sexual maturation has been presented as a significant metabolic risk period for children with obesity [4]. Indeed, we previously described the role of S100A4 in IR through a multi-omics approach in children, providing interesting knowledge into the plausible molecular mechanism underlying that association [10].

In this series of 119 pubertal children, we show higher ISM1 levels in children with obesity compared to normal weight and overweight and a robust positive association between ISM1 serum levels and BMI *Z*-score in boys, indicating a putative sexual dimorphism. Furthermore, circulating ISM1 levels are positively associated with fat mass in children, indicating that the higher ISM1 levels observed might be secreted by adipose cells and might exert endocrine effects in other tissues. However, ISM1 levels were not significantly associated with leptin levels in either boys or girls. Leptin is a well-known adipokine mainly produced by adipose tissue in proportion to the amount of fat mass and is involved in the regulation of food intake and glucose and lipid metabolism, among others [22]. Therefore, circulating leptin concentrations are correlated with the total amount of fat mass [23]; however, individuals with obesity exhibit an impaired response to leptin despite their hyperleptinemia, suggesting a state of leptin resistance [24–26]. The positive correlation between ISM1 and fat content but not with leptin raises the possibility that not all the circulating levels are completely derived from adipose cells. In addition, MPO was negatively associated with serum ISM1 levels. Certainly, MPO is the most abundant protein in human neutrophils, playing a major role in inflammation, oxidative stress, lipoprotein oxidation, and atherosclerosis [17, 27]. In addition, MPO-deficient mice are resistant to diet-induced obesity and IR; inhibition of MPO activity in neutrophils decreases diet-induced IR in obese mice, and activation of MPO is associated with the development of obesity and obesity-associated IR [28].

The negative association between ISM1 and MPO suggests that the all-circulating levels of ISM1 in children with obesity might have a non-adipose cell origin. Group comparisons for ISM1 levels also revealed significant results in boys, although no significant changes were observed when comparing extreme experimental conditions in relation to IR (normal weight vs. obese with IR). Jiang et al. (2021) [7] determined that ISM1 signaling is dependent on phosphoinositide 3-kinase (PI3K) and shares downstream phosphorylation targets with insulin signaling, such as AKT, phosphorylated AKT (p-AKT), phosphorylated extracellular signal-regulated kinase 1/2 (p-ERK1/2), and phosphorylated S6 ribosomal protein (p-S6). Outstandingly, ISM1 activates a PI3K–AKT signaling pathway independent of insulin and insulin-like growth factor 1 receptors and is most likely to signal through another, yet to be identified, receptor tyrosine kinase. We found a correlation with obesity but not with HOMA-IR in children, which would point to the adipocyte-secreted protein ISM1 having a direct role in obesity but not in the metabolic status derived from IR. We should also point out that the origin of circulating ISM1 levels might not be adipose cells. Remarkably, while the glucoregulatory function of the novel adipokine is shared with insulin, ISM1 also neutralizes lipid accumulation in the liver by inhibiting de novo lipogenesis, promoting protein synthesis and preventing hepatic steatosis in a diet-induced fatty liver mouse model. Nevertheless, in addition to disturbed hepatic and postprandial lipoprotein metabolism, enhanced triacylglycerol lipolysis in adipocytes and subsequent fatty acid flux to the liver are major determinants of hepatic steatosis [29]. As insulin is a major anti-lipolytic hormone in adipocytes, it is plausible that ISM1 signaling indirectly modulates hepatic lipid accumulation by inhibiting fatty acid release from adipose tissue in mice [29]. Additionally, in the work of Jiang et al. (2021) [7], the therapeutic dosing of recombinant ISM1 improved glucose tolerance to the same degree as metformin, enhanced diabetes in diet-induced obese mice, and ameliorated hepatic steatosis in a diet-induced fatty liver mouse model, establishing that recombinant ISM1 and its derivatives may be explored for therapeutic purposes and may offer certain advantages over current monotherapies. Nonetheless, the observed higher circulating ISM1 levels in pubertal children suggest that ISM1 resistance may be present, as administration of ISM1 into mice with established disease improves glucose and lipid dysfunction in diet-induced obesity. Additionally, we should consider that pubertal children did not show hyperglycemia, which might mask the observed effects of ISM1 in mice.

The strengths of our findings are the relatively high number of recruited children from different centers in the country (Andalucía, Galicia, and Aragón); the novelty of the recently described new adipokine, which has not been reported elsewhere in humans; and the possibility of correlating with several plasma adipokines, inflammation, and cardiovascular risk biomarkers in the sub-cohort. Furthermore, the availability of DNA methylation analysis using the Infinium Methylation EPIC microarray using bead chip technology in all the children population allowed us to observe two enhancer-related CpG sites of ISM1 (cg03304641 and cg14269097) associated with serum levels of ISM1 in children. A recent study revealed a strong negative correlation between DNA methylation and gene expression for *ISM1* in invasive lobular breast cancer (ILBC), which could potentially serve as a biomarker of survival for women with ILBC [30]. However, the reported CpG sites were different from those in our study.

As a limitation, although ISM1 serum levels are associated with BMI *Z*-score, we cannot distinguish the origin of the circulating levels simply because ISM1 is secreted by other non-adipose cells, and a secondary validation population would be needed to confirm these findings.

In conclusion, the circulating levels of the novel insulin-like adipokine ISM1 are significantly higher in pubertal children with obesity and are associated with BMI *Z*-score and fat mass in boys. Furthermore, we reveal two DNA methylations in two-enhancer-related CpG sites of the ISM1 region associated with serum levels of the protein in children with obesity.

## Supplementary Information

Below is the link to the electronic supplementary material.

Supplementary file1 (PDF 645 KB)

## Data Availability

The datasets generated during and/or analyzed during the current study are available from the corresponding author on reasonable request.
