# Supplementary material for: Serum levels of the novel adipokine isthmin-1 are associated with obesity in pubertal boys
Source: World J Pediatr. 2023 Jan 3;19(9):864–72. doi: 10.1007/s12519-022-00665-8 (PMC10423122; doi:10.1007/s12519-022-00665-8)

Supplementary table 1. Correlation matrix for confounders (R^2)

	Sex	Age	Origin	HOMA	BMI Z-score
Sex	1.0000	0.2921	-0.0208	-0.1898	-0.1089
Age	0.2922	1.0000	-0.0427	-0.1092	-0.0688
Origin	-0.0208	-0.0427	1.0000	-0.0839	-0.1454
HOMA	-0.1898	-0.1092	-0.0839	1.0000	0.5160
BMI-Z-score	-0.1089	-0.0688	-0.1454	0.5160	1.0000

Supplementary table 2. General characteristics, anthropometry, biochemical parameters, adipokines and cardiovascular/pro-inflammatory biomarkers in the cross-sectional cohort of 119 Spanish children.

	Normal-weight	Overweight	Obese	P-Value
Age	14.87 [11.22-17.59] NA=0 [a]	14.22 [9.74-16.85] NA=0 [a]	14.33 [9.72-18.07] NA=0 [a]	0.15170492
Sex	Girls=20; Boys=17 NA=0	Girls=19; Boys=17 NA=0	Girls=25; Boys=20 NA=0	0.97257376
Glucose (mg/dL)	85.11 (9.03) NA=0 [a]	85.33 (6.76) NA=0 [a]	85.2 (8.71) NA=0 [a]	0.78038444
TAG (mg/dL)	63.25 (27.5) NA=1 [b]	66.69 (31.29) NA=0 [ab]	85.64 (37.49) NA=1 [a]	0.00101011
HDLc (mg/dL)	56.5 [32-86] NA=1 [a]	47 [26-70] NA=0 [b]	43 [3-107] NA=1 [b]	2.92E-06
AST (U/L)	22.36 (3.82) NA=1 [a]	20.94 (6.27) NA=0 [a]	22 (5.64) NA=1 [a]	0.15850533
ALT (U/L)	13.62 (4.16) NA=0 [b]	16.58 (6.34) NA=0 [ab]	21.11 (8.61) NA=0 [a]	1.22E-05
GGT (U/L)	11 [6-27] NA=1 [a]	13 [1-27] NA=0 [ab]	15 [2-44] NA=1 [b]	0.00117436
Insulin (mU/L)	8.73 (3.52) NA=0 [b]	12.58 (6.13) NA=0 [b]	21.08 (11.52) NA=0 [a]	6.60E-09
HsCRP (mg/L)	0.63 [0.2-30.1] NA=2 [a]	1.78 [0.2-18.4] NA=2 [b]	4.38 [0.2-164.2] NA=0 [b]	2.89E-05
Waist Hip ratio	0.82 [0.54-1.38] NA=0 [a]	0.83 [0.74-1] NA=1 [a]	0.93 [0.75-2.11] NA=1 [b]	8.42E-06
WC Height ratio	0.43 (0.05) NA=0 [c]	0.52 (0.05) NA=1 [b]	0.61 (0.08) NA=1 [a]	1.47E-28
QUICKI	0.35 [0.32-0.44] NA=0 [a]	0.34 [0.29-0.39] NA=0 [b]	0.31 [0.28-0.42] NA=0 [c]	7.70E-08
DBP (mm Hg)	67.16 (12.49) NA=2 [a]	67.93 (8.11) NA=2 [a]	70.44 (11.51) NA=1 [a]	0.29095778
SBP (mm Hg)	105.77 (13.7) NA=2 [b]	111.07 (12.35) NA=2 [ab]	116.7 (15.42) NA=1 [a]	0.01356016
Glucose (mmol/L)	4.72 (0.5) NA=0 [a]	4.74 (0.38) NA=0 [a]	4.73 (0.48) NA=0 [a]	0.78038444
HOMA-IR	1.84 (0.78) NA=0 [b]	2.69 (1.44) NA=0 [b]	4.53 (2.71) NA=0 [a]	5.38E-08

Waist Circumference (cm^2)	70.15 (7.99) NA=0 [c]	83.08 (7.8) NA=1 [b]	99.43 (11.47) NA=1 [a]	2.44E-25
Hip Circumference (cm^2)	85.37 (9.44) NA=0 [c]	97.59 (7.96) NA=1 [b]	108.88 (10.51) NA=1 [a]	7.87E-18
BMI *Z*-score	-0.12 [-1.59-0.78] NA=0 [a]	1.51 [0.75-2.3] NA=0 [b]	2.9 [2.03-5.82] NA=0 [c]	2.89E-22
sICAM1 (mg/L)	0.07 [0.04-3.31] NA=0 [a]	0.1 [0.06-2.03] NA=0 [b]	0.1 [0.06-1.72] NA=0 [b]	0.00219134
MPO (µg/ L)	23.5 [0.8-196.36] NA=8 [a]	33.63 [0.88-1077.71] NA=3 [a]	32.47 [0.2-744.47] NA=2 [a]	0.31811663
P Selectin (µg/L)	42.17 (12.27) NA=0 [b]	47.1 (15.59) NA=0 [ab]	56.13 (20.87) NA=1 [a]	0.00630924
Adiponectin (mg/L)	13.47 (8.58) NA=0 [a]	12.57 (7.62) NA=0 [a]	8.35 (5.26) NA=0 [b]	0.00722112
Resistin (µg/L)	18.12 [9.19-44.31] NA=0 [a]	18.97 [7.82-56.01] NA=0 [a]	21.18 [10.91-77.23] NA=0 [a]	0.07726358
tPAI1 (µg/L)	8.95 (9.75) NA=0 [b]	16.67 (11.35) NA=0 [b]	25.25 (14.54) NA=0 [a]	1.40E-06
IL6 (ng/L)	8.28 [0.16-173.01] NA=9 [a]	2.89 [0.03-223.84] NA=17 [a]	3.67 [0.12-178.99] NA=8 [a]	0.14984527
IL8 (ng/L)	2.58 [0.56-27.33] NA=0 [a]	2.01 [0.27-30.27] NA=0 [a]	2.29 [0.13-22.8] NA=0 [a]	0.37108233
MCP1 (ng/L)	110.82 (39.87) NA=0 [a]	100.26 (48.49) NA=0 [a]	107.32 (39.99) NA=0 [a]	0.55119851
TNFα (ng/L)	2.73 (0.76) NA=0 [a]	2.57 (1.02) NA=0 [a]	2.66 (0.78) NA=0 [a]	0.71217629
Leptin (ng/L)	3.93 [0.19-21.63] NA=0 [a]	10.7 [1.53-41.58] NA=0 [b]	14.64 [6.42-37.32] NA=0 [c]	2.87E-10
ISM1 (ng/mL)	12.36 [3.9-32.55] NA=0 [a]	13.79 [4.81-31.4] NA=0 [a]	16.77 [6.21-51.7] NA=0 [a]	0.06447697

Data are expressed as mean (standard deviation) or median [min-max] if not normally distributed. One-way ANOVA, Kruskal-Wallis and the Welch test were employed to assess group differences. Distributions within the same row with unlike superscript letters were significantly different ($p < 0.05$) according to Pairwise-t-tests, pairwise Mann–Whitney U-tests and Dunn tests. Childhood obesity was defined according to Cole et al. (2000). Abbreviations: ALR, adiponectin leptin ratio; Apo B, apolipoprotein B; Apo A, apolipoprotein A; BMI, body mass index; DBP, diastolic blood pressure; HC, hip circumference; HDL-c, high-density lipoproteins-cholesterol; HOMA-IR, homeostasis model assessment for insulin resistance; hsCRP, high-sensitivity C reactive protein; IL, interleukin; IR, insulin resistance; ISM1, isthmin-1; LDL-c, low-density lipoproteins-cholesterol; MCP-1, monocyte chemoattractant protein 1; MPO, myeloperoxidase; OB, obesity; PAI-1, plasminogen activator inhibitor-1; QUICKI, quantitative insulin sensitivity check index; SBP, systolic blood pressure; sICAM, soluble intercellular cell adhesion molecule-1; TAG, triacylglycerols; THS; Thyroid-Stimulating Hormone; TNF-α, tumor necrosis factor-alpha; WC, waist circumference. NA: non-available value.

Supplementary table 3. List of Mapped CpG sites in the ISM1 domain.

chr	pos	strand	CpG name	Probe_rs	Probe_maf	UCSC_RefGene_Group	DNase_Hypersensitivity_NAME	DNase_Hypersensitivity_Evidence_Count	TFBS_NAME	TFBS_Evidence_Count	Methyl450_Loci
chr20	13200939	+	cg25796439	NA	NA	TSS1500	chr20:13200665-13201130	3			TRUE
chr20	13200992	+	cg21561970	NA	NA	TSS1500	chr20:13200665-13201130	3			TRUE
chr20	13201650	+	cg15015034	NA	NA	TSS1500	chr20:13201440-13201875	3			TRUE
chr20	13201214	+	cg11155707	rs35907305	0,297039	TSS1500	chr20:13201165-13201435	3			TRUE
chr20	13201655	+	cg03172651	NA	NA	TSS1500	chr20:13201440-13201875	3			TRUE
chr20	13200982	+	cg26322248	NA	NA	TSS1500	chr20:13200665-13201130	3			TRUE
chr20	13201225	+	cg03725761	rs35907305	0,297039	TSS1500	chr20:13201165-13201435	3			TRUE
chr20	13201662	+	cg27427234	NA	NA	TSS1500	chr20:13201440-13201875	3			TRUE
chr20	13202437	+	cg10378364	NA	NA	1stExon	chr20:13202025-13202550	3			TRUE
chr20	13200944	+	cg14060111	NA	NA	TSS1500	chr20:13200665-13201130	3			TRUE
chr20	13202476	-	cg04920227	NA	NA	1stExon	chr20:13202025-13202550	3			TRUE
chr20	13280130	+	cg02962406	NA	NA	3'UTR			chr20:13279440-13280456	3	TRUE
chr20	13200973	+	cg06262436	NA	NA	TSS1500	chr20:13200665-13201130	3			TRUE
chr20	13201342	+	cg23504092	NA	NA	TSS1500	chr20:13201165-13201435	3			TRUE
chr20	13280201	+	cg19727767	NA	NA	3'UTR			chr20:13279440-13280456	3	TRUE
chr20	13201844	-	cg12741255	rs71330293	0,296533	TSS1500	chr20:13201440-13201875	3			TRUE
chr20	13200954	+	cg12664209	NA	NA	TSS1500	chr20:13200665-13201130	3			TRUE
chr20	13201328	+	cg15577927	NA	NA	TSS1500	chr20:13201165-13201435	3			TRUE
chr20	13202222	+	cg20770339	NA	NA	TSS200	chr20:13202025-13202550	3			TRUE
chr20	13200969	+	cg02145932	NA	NA	TSS1500	chr20:13200665-13201130	3			TRUE

chr20	13201829	-	cg25178765	rs71330293	0,296533	TSS1500	chr20:13201440-13201875	3			TRUE
chr20	13202225	+	cg20081364	NA	NA	TSS200	chr20:13202025-13202550	3			TRUE
chr20	13201551	+	cg06567525	NA	NA	TSS1500	chr20:13201440-13201875	3			TRUE
chr20	13200980	+	cg09491991	NA	NA	TSS1500	chr20:13200665-13201130	3			TRUE
chr20	13200931	+	cg04432319	NA	NA	TSS1500	chr20:13200665-13201130	3			TRUE
chr20	13253578	-	cg11466857	NA	NA	Body					
chr20	13246774	-	cg22470917	rs7269783	0,059433	Body	chr20:13246645-13246895	3			
chr20	13251901	-	cg04049812	NA	NA	Body	chr20:13251840-13252255	3			
chr20	13228392	+	cg01440309	NA	NA	Body	chr20:13227965-13228730	3			
chr20	13202489	-	cg06355010	NA	NA	1stExon	chr20:13202025-13202550	3			TRUE
chr20	13203343	+	cg12235430	NA	NA	Body					
chr20	13226652	-	cg21903043	rs140456570	0,0133	Body					
chr20	13280181	+	cg16294620	NA	NA	3'UTR			chr20:13279440-13280456	3	TRUE
chr20	13243701	-	cg02885188	NA	NA	Body	chr20:13243460-13243955	3			
chr20	13211902	+	cg14269097	NA	NA	Body	chr20:13211665-13211950	3			
chr20	13226121	-	cg18950540	NA	NA	Body	chr20:13226080-13226250	3			
chr20	13201670	+	cg03623378	NA	NA	TSS1500	chr20:13201440-13201875	3			TRUE
chr20	13201353	+	cg19118558	NA	NA	TSS1500	chr20:13201165-13201435	3			TRUE
chr20	13279847	+	cg25787377	NA	NA	Body			chr20:13279440-13280456	3	TRUE
chr20	13279946	+	cg19647607	NA	NA	Body			chr20:13279440-13280456	3	TRUE
chr20	13243555	-	cg24256058	NA	NA	Body	chr20:13243460-13243955	3			
chr20	13279761	+	cg20777315	NA	NA	Body			chr20:13279440-13280456	3	TRUE
chr20	13277967	+	cg24488317	NA	NA	Body	chr20:13277725-13278235	3			TRUE
chr20	13205832	+	cg25411902	NA	NA	Body					TRUE
chr20	13227047	+	cg03304641	rs111284324	0,035224	Body					
chr20	13261553	+	cg13735403	NA	NA	Body	chr20:13261540-13261770	3			

chr20	13201349	+	cg25384906	NA	NA	TSS1500	chr20:13201165-13201435	3	TRUE
chr20	13201205	-	cg19938101	rs34502335	0,000455	TSS1500	chr20:13201165-13201435	3	TRUE
chr20	13201216	+	cg25606624	rs35907305	0,297039	TSS1500	chr20:13201165-13201435	3	TRUE
chr20	13201193	-	cg12448198	rs34502335	0,000455	TSS1500	chr20:13201165-13201435	3	TRUE
chr20	13267421	+	cg09495909	NA	NA	Body	chr20:13267320-13267535	3	

Figure 1. Correlation matrix for confounders (R^2)

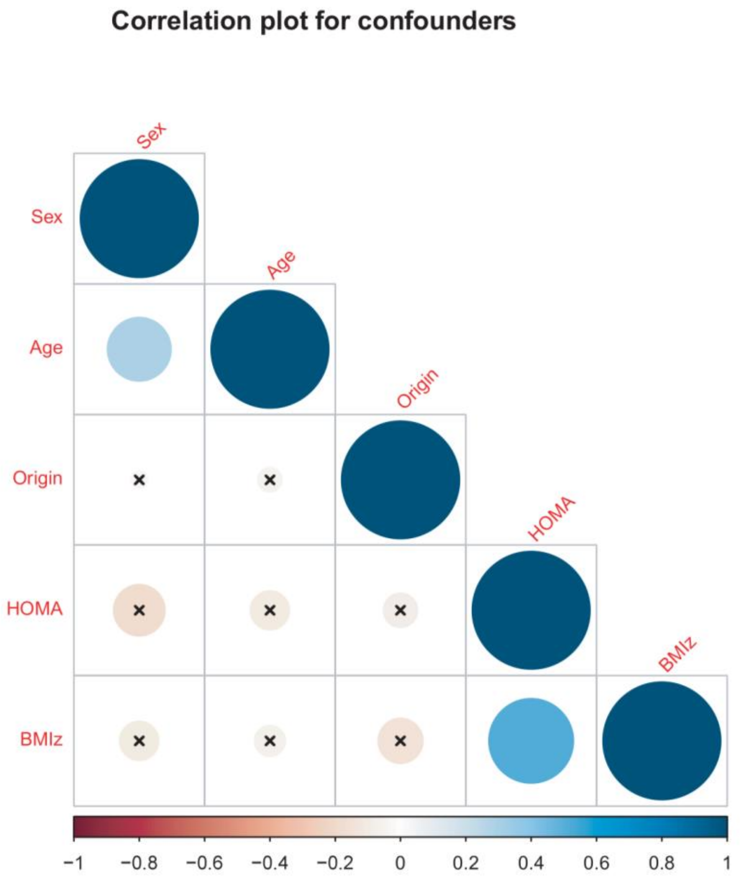

Figure 2. Multiple linear regressions were employed with M values as independent variables and each outcome as the dependent variable

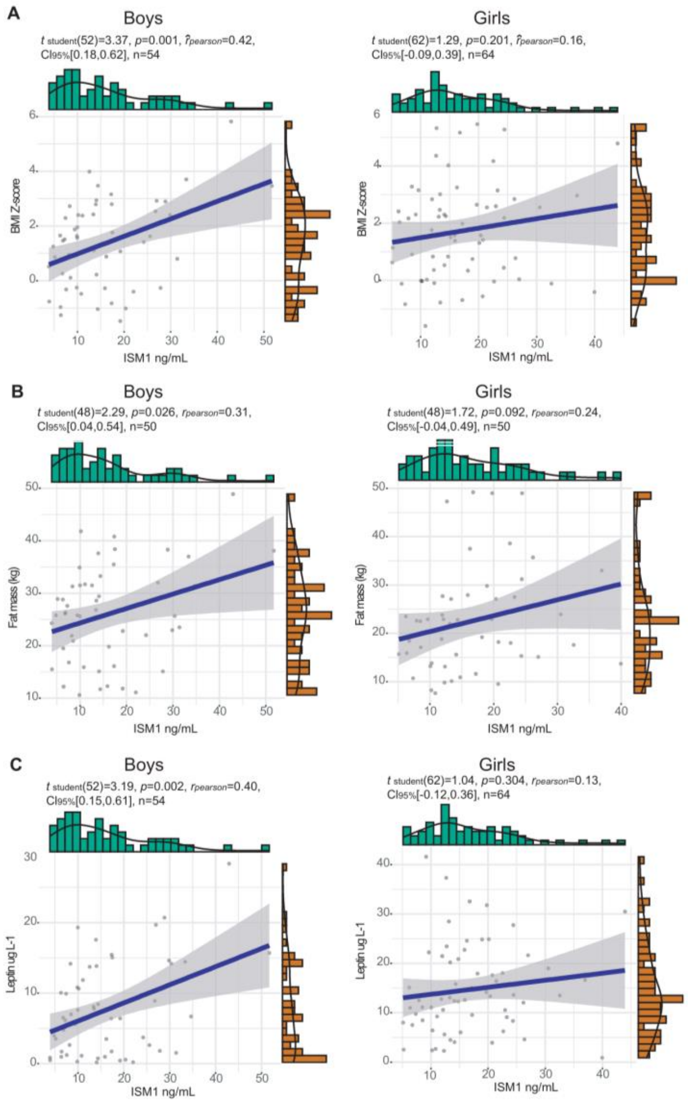

Figure 3. Cross-sectional associations between ISM1 DNA methylation status and ISM1 serum levels (ng/mL).

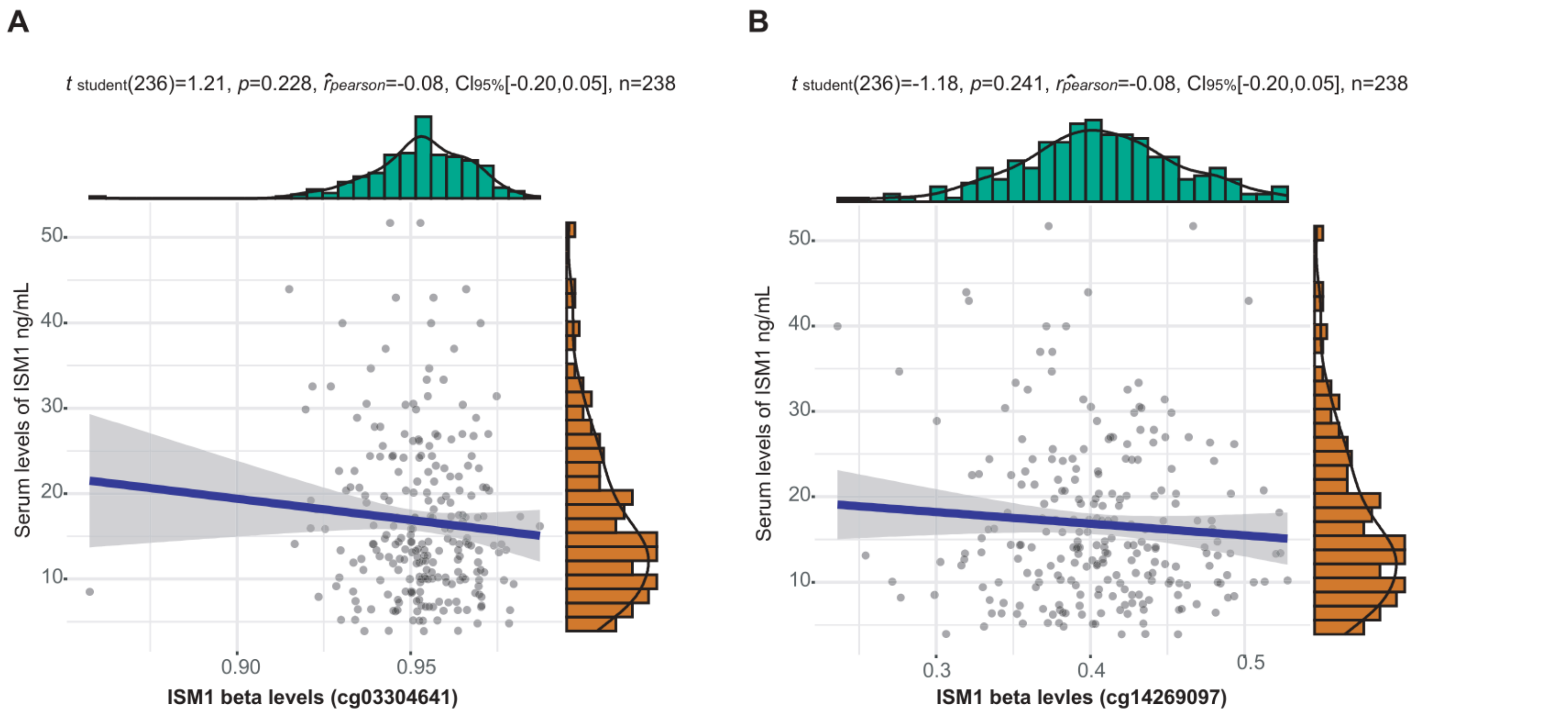

Supplement: Supplementary file 1 — Supplementary file1 (PDF 645 KB) [file 12519_2022_665_MOESM1_ESM.pdf]
